# CSF α-synuclein seed amplification kinetic profiles are associated with cognitive decline in Parkinson’s disease

**DOI:** 10.1038/s41531-023-00627-5

**Published:** 2024-01-20

**Authors:** Kathrin Brockmann, Stefanie Lerche, Simone Baiardi, Marcello Rossi, Isabel Wurster, Corinne Quadalti, Benjamin Roeben, Angela Mammana, Milan Zimmermann, Ann‑Kathrin Hauser, Christian Deuschle, Claudia Schulte, Inga Liepelt-Scarfone, Thomas Gasser, Piero Parchi

**Affiliations:** 1 Department of Neurodegeneration, Center of Neurology, Hertie Institute for Clinical Brain Research, German Center for Neurodegenerative Diseases, University of Tuebingen, Hoppe Seyler‑Strasse 3, 72076 Tuebingen, Germany; 2 German Center for Neurodegenerative Diseases, University of Tuebingen, Tuebingen, Germany; 3 IRCCS Istituto delle Scienze Neurologiche di Bologna, Via Altura 1/8, 40139 Bologna, Italy; 4 Department of Biomedical and Neuromotor Sciences (DiBiNeM), University of Bologna, Bologna, Italy; 5 Edmond J. Safra Fellow in Movement Disorders, Tuebingen, Germany; 6 Present Address: Department of Pharmacy and Biotechnology, University of Bologna, 40126 Bologna, Italy

**Keywords:** Parkinson's disease, Prognostic markers

## Abstract

Seed amplification assays have been implemented in Parkinson’s disease to reveal disease-specific misfolded alpha-synuclein aggregates in biospecimens. While the assays’ qualitative dichotomous seeding response is valuable to stratify and enrich cohorts for alpha-synuclein pathology in general, more quantitative parameters that are associated with clinical dynamics of disease progression and that might potentially serve as exploratory outcome measures in clinical trials targeting alpha-synuclein would add important information. To evaluate whether the seeding kinetic parameters time required to reach the seeding threshold (LAG phase), the peak of fluorescence response (Imax), and the area under the curve (AUC) are associated with clinical trajectories, we analyzed LAG, Imax, and AUC in relation to the development of cognitive decline in a longitudinal cohort of 199 people with Parkinson’s disease with positive CSF alpha-synuclein seeding status. Patients were stratified into tertiles based on their individual CSF alpha-synuclein seeding kinetic properties. The effect of the kinetic parameters on longitudinal development of cognitive impairment defined by MoCA ≤25 was analyzed by Cox-Regression. Patients with a higher number of positive seeding replicates and tertile groups of shorter LAG, higher Imax, and higher AUC showed a higher prevalence of and a shorter duration until cognitive impairment longitudinally (3, 6, and 3 years earlier with *p* ≤ 0.001, respectively). Results remained similar in separate subgroup analyses of patients with and without *GBA* mutation. We conclude that a more prominent alpha-synuclein seeding kinetic profile translates into a more rapid development of cognitive decline.

## Introduction

The clinicopathological heterogeneity in Parkinson’s disease (PD) and the limitation of current clinical diagnostic criteria, especially at disease onset, highlight the need for pathology-specific biomarkers. Misfolded alpha-synuclein (α-Syn), the hallmark of the typical Lewy body pathology, is a lead candidate based on its crucial role in disease pathophysiology. With disease-modifying compounds such as monoclonal antibodies or active vaccination targeting α-Syn currently tested in clinical trials, patient stratification according to α-Syn-specific enrichment strategies is a much-needed prerequisite. Recently, highly sensitive seed amplification assays (SAA) exploiting the seeding capacities of prion and prion-like proteins as an amplification strategy to reveal minute amounts of disease-specific protein aggregates in CSF and other accessible biomaterials^1–7^ have shown high sensitivity (86–96%) and specificity (83–100%) for sporadic PD and dementia with Lewy bodies (DLB) compared to healthy individuals^8,9^. Using SAA in CSF, it was shown that people with PD and DLB carrying severe mutations in the gene *glucocerebrosidase* (*GBA*) manifest a higher prevalence of α-Syn seeding (93% and 100%) compared to other genetic forms with known variable (*LRRK2*, 78% positive seeding) or even sparse Lewy body pathology (bi-allelic *Parkin*, *PINK1*, 0% positive seeding)^10–12^. Thereby, SAAs might serve as a specific biomarker of Lewy body associated α-Syn pathology. While the assays’ qualitative dichotomous seeding response (positive/negative seeding) is valuable to stratify and enrich cohorts for α-Syn pathology in general, more quantitative parameters that are associated with dynamics of disease progression and phenotypical trajectories and that could potentially serve as exploratory outcome measures targeting α-Syn would add important information. In this context, the kinetic parameters of the SAA reaction, such as the LAG phase (LAG) (time required to reach the seeding threshold), the peak of the fluorescence response (Imax), and the area under the curve (AUC) of the fluorescence signal are candidates to be evaluated in individuals who show positive α-Syn seeding. First pilot data show that shorter LAG and higher AUC and Imax are associated with higher UPDRS-III and lower MoCA scores in PD and that people with PD carrying severe mutations in the *GBA* gene present more prominent CSF α-Syn seeding kinetic profiles than PD patients without *GBA* mutation^10,12,13^. Based on these findings, we hypothesize that prominent CSF α-Syn seeding kinetic profiles are associated with faster progression to clinical disease milestones such as cognitive impairment. To further evaluate this hypothesis with longitudinal clinical trajectories, we analyzed a large cohort of PD patients with a positive CSF α-Syn seeding status.

## Results

### Cross-sectional analysis

Of the 231 analyzed patients, 199 showed positive CSF α-Syn seeding, 30 were non-seeders, and two were unclear (1 out of 4 runs). The non-seeders and unclear seeders were excluded from analyses. For details and a comparison of non-seeders versus seeders, we kindly refer to our previous manuscript^10^.

Correlation analyses showed that higher numbers of positive seeding replicates (0.215; *p* = 0.003), shorter LAG phase (−0221.; *p* = 0.002), as well as higher Imax (0.186; *p* = 0.009) and higher AUC (0.209; *p* = 0.003) were associated with the presence of cognitive impairment. Similarly, higher numbers of positive seeding replicates (−0.346; *p* ≤ 0.001), shorter LAG phase (0.171; *p* = 0.018), as well as higher Imax (−0.192; *p* = 0.008), and higher AUC (−0.193; *p* = 0.008) were associated with lower MoCA scores.

Higher numbers of positive seeding replicates (0.151; *p* = 0.037), shorter LAG phase (−0.152; *p* = 0.026), as well as higher Imax (0.148; *p* = 0.040), and higher AUC (0.156; *p* = 0.031) were associated with higher UPDRS-III scores.

Higher numbers of positive seeding replicates were associated with age (0.289; *p* ≤ 0.001) and with disease duration (0.260; *p* ≤ 0.001). LAG phase, Imax, or AUC were not associated with age or disease duration.

Group comparison stratified by the number of positive CSF α-Syn seeding replicates showed that:

1. Patients with 4 out of 4 positive CSF α-Syn seeding replicates presented with a higher prevalence of cognitive impairment (58% vs. 40% vs. 38%; *p* = 0.050), lower mean MoCA scores (24 vs. 27 vs. 26; *p* = 0.022), a shorter mean LAG phase (20 h vs. 22 h vs. 23 h; *p* ≤ 0.001), a higher mean Imax (72 vs. 67 vs. 61; *p* = 0.008) and a higher mean AUC (806 vs. 686 vs. 598; *p* ≤ 0.001) compared to the groups with 3 and with 2 positive CSF α-Syn seeding replicates out of 4. Also, *GBA* mutation carrier status was highest in the group with 4 out of 4 positive CSF α-Syn seeding replicates compared to the groups with 3 and with 2 positive CSF α-Syn seeding replicates (50% vs. 36% vs. 18%; *p* = 0.018). Mean levels of Amyloid-ß_1_42_, total-Tau, phospho-Tau181, Neurofilament light chain, and prevalence of a CSF Alzheimer’s signature did not differ significantly between groups. For details, see Table 1.

**Table 1:** Demographic, clinical, and CSF characteristics stratified by number of positive seeding replicates.

Number of positive seeding replicates out of 4	2	3	4	*p* value
Male sex %	82	59	69	0.150
Age, years	59 ± 8	62 ± 9	66 ± 9 ^**, §§^	≤0.001
Age at onset, years	55 ± 9	56 ± 10	59 ± 9	0.050
Disease duration, years	4 ± 3	6 ± 4	7 ± 5 *	0.009
H&Y	2.1 ± 0.6	2.2 ± 0.6	2.0 ± 0.6	0.325
H&Y ≥ 2.5%	12	8	22	0.058
UPDRS-III	22 ± 6	24 ± 11	27 ± 11	0.497
Montreal cognitive assessment	26 ± 5	27 ± 3	24 ± 4 ^§§§^	0.022
Cognitive impairment				
At baseline %	25	17	44	0.002
New during study %	17	28	25	0.731
Cognitive impairment anytime %	38	40	58	0.050
BDI-II	9 ± 7	8 ± 7	9 ± 6	0.321
LEDD	510 ± 357	506 ± 417	517 ± 324	0.631
Amyloidβ_1–42_ [pg/ml]	754 ± 195	724 ± 216	706 ± 287	0.637
total-Tau [pg/ml]	183 ± 71	213 ± 108	258 ± 142	0.557
phospho181-Tau [pg/ml]	33 ± 11	38 ± 13	42 ± 16	0.392
AD profile %	0	0	0.8	0.763
Neurofilament light chain [pg/ml]	851 ± 1040	753 ± 406	992 ± 947	0.577
total α-synuclein [pg/ml]	598 ± 188	528 ± 239	586 ± 267	0.937
LAG, h	23 ± 3	22 ± 3	20 ± 3^***, §§§^	≤0.001
Imax	61 ± 13	67 ± 13	72 ± 13**	0.008
AUC	598 ± 188	686 ± 235	806 ± 225 ^***,§§^	≤0.001
Patients with GBA mutation	18%	36%	50%	0.018
Patients with LRKK2 mutation	0%	4%	4%	0.712
Patients with PRKN mutation	7%	6%	5%	0.903

*BDI-II* Becks depression Inventory version II, *H&Y* Hoehn and Yahr, *LEDD* Levodopa Equivalent Daily Dose, *UPDRS-III* Unified Parkinson Disease rating Scale part III, *AD* Alzheimer’s Disease CSF profile defined by the National Institute on Aging (NIA)-Alzheimer’s Association (AA) Research Framework.

Data are presented as mean and standard deviation.

Post-hoc Bonferroni subgroup comparison 2 vs. 4 levels of significance: *p < 0.05; **p < 0.01; ***p ≤ 0.001.

Post-hoc Bonferroni subgroup comparison 3 vs. 4 levels of significance: ^§^*p* < 0.05; ^§§^*p* < 0.01; ^§§§^*p* ≤ 0.001.

Separate subgroup analysis in PD-wildtype (without pathogenic mutation in the genes *GBA*, *LRRK2*, *PRKN*, *PINK*) and PD-GBA (PD with *GBA* mutation) confirmed results from the total PD group in both subcohorts with lower mean MOCA (PD-wildtype: 25 vs. 27 vs. 27, *p* = 0.031; PD_GBA: 23 vs. 27 vs. 23; *p* = 0.016), shorter LAG phase (PD-wildtype: 21 h vs. 22 h vs. 22 h, *p* = 0.034; PD_GBA: 19 vs. 21 vs. 23; *p* = 0.007), and higher AUC (PD-wildtype: 770 vs. 658 vs. 605, *p* = 0.014; PD_GBA: 837 vs. 733 vs. 569; *p* = 0.040) in patients with 4 out of 4 positive CSF α-Syn seeding replicates compared to the groups with 3 and with 2 positive CSF α-Syn seeding replicates (Supplementary Table 1).

Group comparison stratified by CSF α-Syn seeding kinetic tertiles showed that:

1. Patients in the lowest tertile of the LAG (shortest LAG phase) presented with the highest prevalence of cognitive impairment (61% vs. 54% vs. 41%; *p* = 0.05) and of *GBA* mutation carrier status (56% vs. 40% vs. 34%; *p* = 0.032) compared to the mid and highest (longest) LAG tertile groups. Moreover, they had lower mean CSF levels of phospho181-tau (36 vs. 43 vs. 41, *p* = 0.031) compared to patients in the mid LAG tertile (longest LAG phase). Prevalence of a CSF Alzheimer’s signature did not differ significantly between groups. For details, see Table 2.

**Table 2:** Demographic, clinical, and CSF characteristics stratified by LAG Tertile groups.

LAG	Highest tertile (longest LAG)	Mid-tertile	Lowest-tertile (shortest LAG)	*p* value
Male sex %	73	58	71	0.115
Age, years	64 ± 10	65 ± 10	64 ± 8	0.885
Age at onset, years	58 ± 10	58 ± 9	57 ± 9	0.496
Disease duration, years	6 ± 4	7 ± 5	7 ± 5	0.182
H&Y	2.0 ± 0.6	2.2 ± 0.6	2.1 ± 0.6	0.205
H&Y ≥ 2.5%	12	21	18	0.350
UPDRS-III	24 ± 10	25 ± 10	28 ± 12	0.169
Montreal cognitive assessment	26 ± 4	25 ± 5	25 ± 4	0.342
Cognitive impairment				
At baseline %	29	37	39	0.468
New during study %	15	27	36	0.089
Cognitive impairment anytime %	41	54	61	0.050
BDI-II	9 ± 6	8 ± 7	9 ± 7	0.773
LEDD	517 ± 324	506 ± 417	510 ± 357	0.986
Amyloidβ_1–42_ [pg/ml]	713 ± 233	738 ± 268	695 ± 286	0.654
Total-Tau [pg/ml]	260 ± 180	245 ± 105	214 ± 86	0.121
Phospho181-Tau [pg/ml]	41 ± 17	43 ± 16°	36 ± 12	0.031
AD Profile %	1.5	0	0	0.377
Neurofilament light chain [pg/ml]	1008 ± 1284	925 ± 504	804 ± 458	0.389
Total α-synuclein [pg/ml]	575 ± 246	610 ± 311	506 ± 192	0.064
Patients with GBA mutation	34%	40%	56%	0.032
Patients with LRKK2 mutation	2%	5%	5%	0.543
Patients with PRKN mutation	8%	5%	3%	0.486

*BDI-II* Becks depression Inventory version II, *H&Y* Hoehn and Yahr, *LEDD* Levodopa Equivalent Daily Dose, *UPDRS-III* Unified Parkinson Disease rating Scale part III. *AD* Alzheimer’s Disease CSF profile defined by the National Institute on Aging (NIA)-Alzheimer’s Association (AA) Research Framework.

Data are presented as mean and standard deviation.

Post-hoc Bonferroni subgroup comparison for continuous data lowest vs. mid-tertile with levels of significance: °*p* < 0.05; °°*p* < 0.01; °°°*p* ≤ 0.001.
2. Patients in the highest and mid Imax tertile presented with the highest prevalence of cognitive impairment (62% vs. 60% vs. 33%, *p* ≤ 0.001) compared to patients in the lowest tertile. Prevalence of a CSF Alzheimer’s signature did not differ significantly between groups. For details, see Table 3.

**Table 3:** Demographic, clinical, and CSF characteristics stratified by Imax Tertile groups.

Imax	Lowest tertile (lowest Imax)	Mid-tertile	Highest Tertile (highest Imax)	*p* value
Male sex %	67	72	63	0.571
Age, years	63 ± 9	64 ± 11	66 ± 8	0.097
Age at onset, years	57 ± 9	57 ± 10	59 ± 8	0.347
Disease duration, years	6 ± 4	7 ± 5	7 ± 5	0.243
H&Y	1.9 ± 0.6	2.2 ± 0.6	2.2 ± 0.7*	0.038
H&Y ≥ 2.5%	9	20	22	0.091
UPDRS-III	24 ± 10	26 ± 12	27 ± 11	0.176
Montreal Cognitive Assessment	27 ± 3	24 ± 5°°	24 ± 4**	≤0.001
Cognitive impairment				
At baseline %	19	48	38	0.002
New during study %	17	21	39	0.047
Cognitive impairment anytime %	33	60	62	≤0.001
BDI-II	9 ± 7	10 ± 7	8 ± 7	0.535
LEDD	486 ± 406	597 ± 353	451 ± 321	0.060
Amyloidβ_1–42_ [pg/ml]	756 ± 244	703 ± 271	685 ± 270	0.264
Total-Tau [pg/ml]	251 ± 160	251 ± 136	218 ± 87	0.259
Phospho181-Tau [pg/ml]	41 ± 15	42 ± 17	38 ± 13	0.355
AD Profile %	1.5	0	0	0.377
Neurofilament light chain [pg/ml]	948 ± 1282	937 ± 508	858 ± 455	0.803
Total α-synuclein [pg/ml]	601 ± 271	571 ± 245	524 ± 254	0.221
Patients with GBA mutation	34%	50%	46%	0.165
Patients with LRKK2 mutation	0%	5%	6%	0.148
Patients with PRKN mutation	10%	2%	5%	0.164

*BDI-II* Becks depression Inventory version II, *H&Y* Hoehn and Yahr, *LEDD* Levodopa Equivalent Daily Dose, *UPDRS-III* Unified Parkinson Disease Rating Scale part III.

Data are presented as mean and standard deviation. *AD* Alzheimer’s Disease CSF profile defined by the National Institute on Aging (NIA)-Alzheimer’s Association (AA) Research Framework.

Post-hoc Bonferroni subgroup comparison for continuous data lowest vs. mid-tertile with levels of significance: °*p* < 0.05; °°*p* < 0.01; °°°*p* ≤ 0.001.

Post-hoc Bonferroni subgroup comparison for continuous data lowest vs. highest Tertile with levels of significance: *p < 0.05; **p < 0.01; ***p ≤ 0.001.
3. Patients in the highest and mid AUC tertile presented with the highest prevalence of cognitive impairment (55% vs. 64% vs. 36%; *p* = 0.005) and of *GBA* mutation carrier status (52% vs. 47% vs. 32%; *p* = 0.050) compared to the lowest AUC tertile group. The prevalence of a CSF Alzheimer’s signature did not differ significantly between groups. For details, see Table 4.

**Table 4:** Demographic, clinical, and CSF characteristics stratified by AUC Tertile groups.

Area under the curve (AUC)	Lowest tertile (lowest AUC)	Mid-tertile	Highest tertile (highest AUC)	*p* value
Male sex %	70	63	69	0.670
Age, years	64 ± 9	64 ± 10	65 ± 8	0.801
Age at onset, years	58 ± 10	57 ± 10	58 ± 9	0.912
Disease duration, years	6 ± 4	7 ± 5	7 ± 5	0.167
H&Y	2.0 ± 0.6	2.2 ± 0.6	2.1 ± 0.7	0.218
H&Y ≥ 2.5%	11	22	19	0.216
UPDRS-III	24 ± 10	25 ± 11	27 ± 12	0.271
Montreal Cognitive Assessment	26 ± 4	24 ± 4°	25 ± 4	0.027
Cognitive impairment				
At baseline %	23	50	32	0.006
New during study %	16	28	33	0.153
Cognitive impairment anytime %	36	64	55	0.005
BDI-II	10 ± 7	8 ± 7	9 ± 7	0.391
LEDD	508 ± 335	515 ± 390	510 ± 375	0.994
Amyloidβ_1–42_ [pg/ml]	726 ± 225	724 ± 280	696 ± 282	0.756
total-Tau [pg/ml]	257 ± 165	251 ± 135	213 ± 77	0.103
phospho181-Tau [pg/ml]	42 ± 15	42 ± 18	37 ± 11	0.064
AD Profile %	1.5	0	0	0.369
Neurofilament light chain [pg/ml]	996 ± 1300	934 ± 522	816 ± 448	0.469
total α-synuclein [pg/ml]	602 ± 258	584 ± 300	510 ± 202	0.100
Patients with GBA mutation	32%	47%	52%	0.050
Patients with LRKK2 mutation	3%	5%	3%	0.842
Patients with PRKN mutation	10%	3%	3%	0.194

*BDI-II* Becks Depression Inventory version II, *H&Y* Hoehn and Yahr *LEDD* Levodopa Equivalent Daily Dose, *UPDRS-III* Unified Parkinson Disease Rating Scale part III. *AD* Alzheimer’s Disease CSF profile defined by the National Institute on Aging (NIA)-Alzheimer’s Association (AA) Research Framework.

Data are presented as mean and standard deviation.

Post-hoc Bonferroni subgroup comparison for continuous data lowest vs. mid tertile with levels of significance: °*p* < 0.05; °°*p* < 0.01; °°°*p* ≤ 0.001.

Separate subgroup analysis in PD-wildtype and PD-GBA showed comparable results for LAG, Imax, and AUC, indicating that findings in the total PD group are not primarily driven by *GBA* mutation status, Supplementary Tables 2–4.

### Longitudinal analysis

Of the 199 analyzed patients, 68 had cognitive impairment at baseline. Of the 131 without cognitive impairment at baseline, 116 (89%) were followed longitudinally for a mean study duration of 6 years. Of these, 32 patients (28%) developed cognitive impairment during study follow-up. Patients developing cognitive impairment during follow-up reached this milestone at a mean study duration of 6 years with a mean disease duration of 10 years, whereas patients not manifesting cognitive impairment were followed up for a mean study duration of 5 years until a mean disease duration of 11 years (study duration *p* = 0.055; disease duration *p* = 0.760).

We found that:

1. Patients in the lowest tertile of the LAG phase (shortest LAG phase) more often developed cognitive impairment during the study compared to patients in the highest LAG tertile (longest LAG phase) (36% vs. 15%, *p* = 0.027).
2. Patients in the highest tertile of Imax developed more often cognitive impairment during study when compared to patients in the lowest tertile (39% vs 17%, *p* = 0.033).

**Fig. 1:**
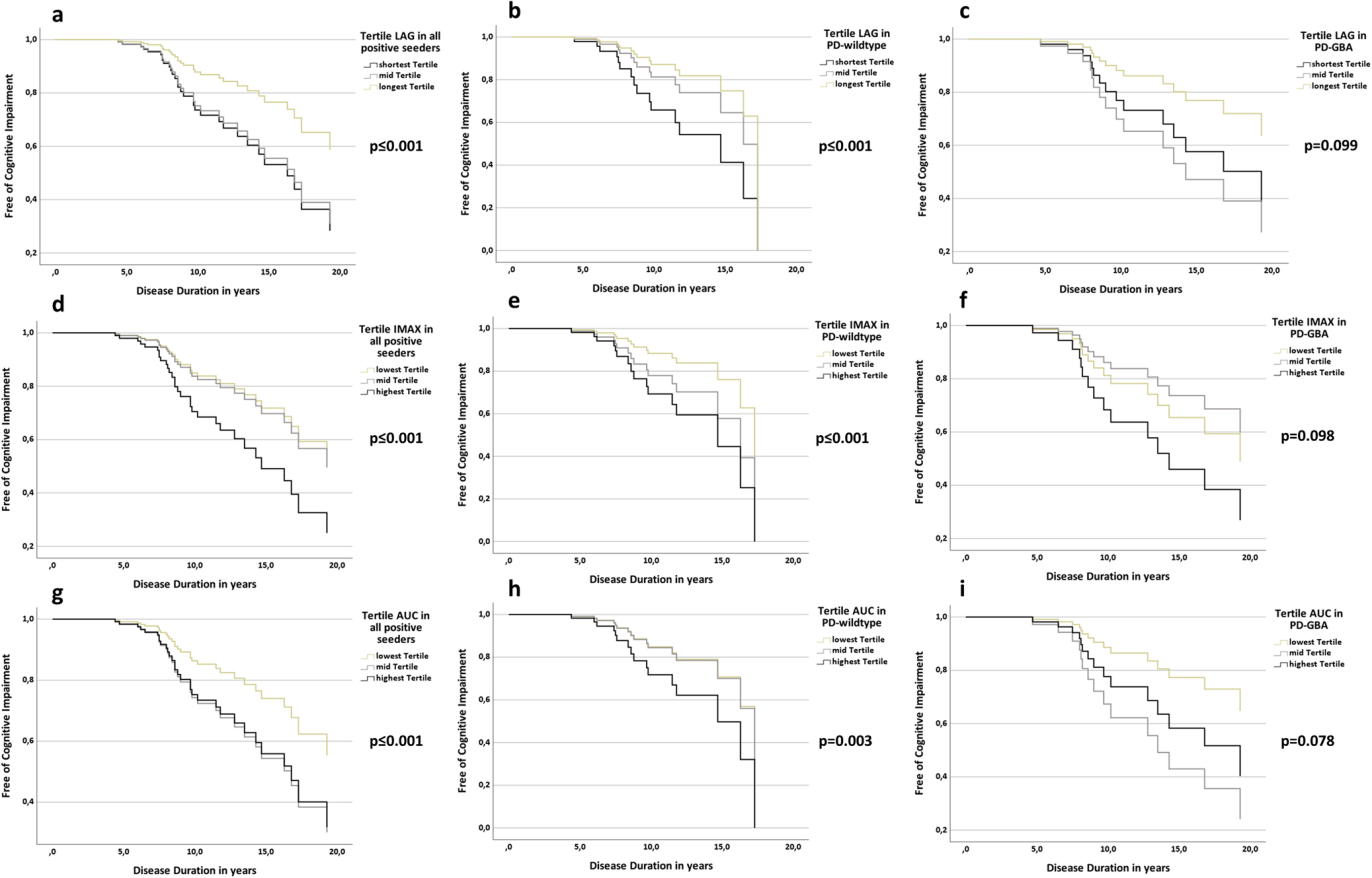
Cox regression analyses modeling the interaction of baseline MoCA and LAG, Imax, AUC Tertiles on the longitudinal development until cognitive impairment in the total cohort as well as in PD-wildtype and PD-GBA separately. **a** Longitudinal development until cognitive impairment in the total cohort based on LAG tertiles. **b** Longitudinal development until cognitive impairment in PD-wildtype based on LAG tertiles. **c** Longitudinal development until cognitive impairment in PD-GBA based on LAG tertiles. **d** Longitudinal development until cognitive impairment in the total cohort based on Imax tertiles. **e** Longitudinal development until cognitive impairment in PD-wildtype based on Imax tertiles. **f** Longitudinal development until cognitive impairment in PD-GBA based on Imax tertiles. **g** Longitudinal development until cognitive impairment in the total cohort based on AUC tertiles. **h** Longitudinal development until cognitive impairment in PD-wildtype based on AUC tertiles. **i** Longitudinal development until cognitive impairment in PD-GBA based on AUC tertiles.

Cox-Regression analyses modeling the interaction of baseline-MoCA and kinetic tertile groups revealed that shorter LAG, higher Imax, and higher AUC significantly affect the time lapse until the development of cognitive impairment (Fig. 1). Specifically:

1. Fifty percent of patients in the shortest and mid-tertile of LAG reached this milestone after 17 years of mean disease duration compared to 20 years of mean disease duration in patients of the longest tertile (*p* ≤ 0.001).
  Separate subgroup analysis in PD-wildtype and PD-GBA revealed similar results:
  PD-wildtype: the shortest LAG tertile reached cognitive impairment after 15 years compared to 17 years for the mid and longest LAG tertile (*p* ≤ 0.001).
  PD-GBA: the shortest and mid LAG tertile reached cognitive impairment after 14 and 15 years compared to 19 years for the longest LAG tertile (*p* = 0.099).
2. Fifty percent of patients in the highest tertile of Imax reached this milestone after 14 years of mean disease duration compared to 20 years of mean disease duration in patients of the mid and lowest tertile (*p* ≤ 0.001).
  Separate subgroup analysis in PD-wildtype and PD-GBA revealed similar results:
  PD-wildtype: the highest Imax tertile reached cognitive impairment after 15 years compared to 16 and 17 years for the mid and lowest Imax tertile (*p* ≤ 0.001).
  PD-GBA: the mid and highest Imax tertile reached cognitive impairment after 14 and 16 years compared to 20 years for the lowest Imax tertile (*p* = 0.098).
3. Fifty percent of patients in the highest and mid tertile of AUC reached this milestone after 17 years of mean disease duration compared to 20 years of mean disease duration in patients of the lowest tertile (*p* ≤ 0.001).

Separate subgroup analysis in PD-wildtype and PD-GBA revealed similar results:

PD-wildtype: the highest AUC tertile reached cognitive impairment after 15 years compared to 17 years for the mid and lowest Imax tertile (*p* = 0.003).

PD-GBA: the mid and highest AUC tertile reached cognitive impairment after 13 and 15 years compared to 20 years for the lowest Imax tertile (*p* = 0.078).

## Discussion

The present study shows that a higher number of positive replicates and a more prominent α-Syn SAA kinetic profile reflected by shorter LAG, higher Imax, and higher AUC is associated with a higher prevalence of cognitive impairment and with a shorter duration until the development of cognitive impairment longitudinally. Importantly, these characteristics could be detected not only in the total cohort of PD patients with and without mutations in PD-related genes but also in the subanalysis stratified by PD-wildtype and PD-GBA only. Notably, mean CSF levels of Amyloid-β_1–42_, total-Tau, and phospho181-Tau were in the normal range in most cases and did not differ significantly across the different α-Syn SAA replicate and kinetic tertile groups. Accordingly, the prevalence of a CSF Alzheimer’s signature was low (0–2%) and did not differ between the analyzed groups. Thereby, the significant associations we found between α-Syn SAA kinetic profiles and cognitive decline cannot be attributed to concomitant Alzheimer’s disease pathology.

In SAAs, the initially low number of fibrils act as seeds for the amplification of fibril mass to a detectable level. Amplification is achieved by adding monomeric α-Syn as a reactant. The seeds in the sample recruit and convert monomeric α-Syn, which is incorporated into longer fibrils. Shaking the sample induces fragmentation of the elongated fibrils, increasing the number of fibrils and the chance of interactions and conversion. These newly created fibrils again grow by recruiting monomeric α-Syn, perpetuating this cycle. This combination of events is a time- and seed-concentration-dependent evolution, ultimately leading to an explosive increase in fibrils. The amplification is monitored using the fluorescent dye thioflavin-T (ThT). Upon binding to the fibril, ThT becomes strongly fluorescent. The time required to reach a predefined seeding threshold (LAG), the peak of fluorescence intensity (Imax), and the overall fluorescence area (AUC) are quantitative readouts expressing the kinetics of the reaction. Based on evidence indicating that the LAG is inversely associated with the seed concentration in the analyzed biospecimen^14–16^, one might assume that a higher CSF α-Syn seed concentration, consequent to a more pronounced/widespread α-Syn-driven Lewy body pathology and a higher leakage of misfolded α-Syn species from degenerating neurons might result in a more “prominent” CSF α-Syn SAA kinetic profile (short LAG, high Imax, high AUC). Indeed, a recent post-mortem study demonstrated that ventricular CSF α-Syn seeding was positive in 100% of cases with neocortical Lewy body pathology (stage IV). In contrast, only 7% of cases with olfactory bulb (stage I) or brainstem-predominant (stage II) Lewy body pathology were CSF α-Syn seeding positive^17^. Thereby, the CSF α-Syn seeding response seems associated with the staging/distribution of Lewy body pathology. However, associations between α-Syn SAA kinetic profiles and clinical measures of disease severity in rather small PD cohorts have been inconsistent to date. While some studies in CSF reported weak associations between short LAG phase and higher Imax with longer disease duration^9^ and higher MDS-UPDRS-III^9^, other studies found no association^18^. One reasonable explanation for these inconsistent results, currently limiting the “quantitative” utility of α-Syn- AA for CSF, is the substantial variability associated with the different assays. This could be due to the inherent variability of the aggregation process, differences in reagents or procedures, and the relatively low numbers of samples analyzed. Evidence also indicates that the CSF milieu, including the extremely low concentrations of the biomarkers, is a critical component of the SAA reaction that directly affects the kinetic parameters of the reaction. In this regard, it was recently shown that CSF lipoproteins have an inhibitory effect on α-Syn aggregation and CSF SAA reaction^19^. In this context, the large sample cohort analyzed, and the use of highly standardized experimental conditions likely contributed to our finding of a significant association between the assay kinetics and clinical parameters. Therefore, our results, by supporting the value of the α-Syn CSF SAA as a quantitative assay, should encourage further work to improve the assay kinetics’ reproducibility by identifying all variables affecting these parameters to reach the final goal of an improved and standardized assay applicable to the clinical setting.

An alternative approach that might contribute to a reliable quantification of the seed concentration is the end-point dilution. This approach uses the modified Spearman-Karber method to compare seed concentrations by estimating the number of samples containing enough seeding activity to yield positive responses in 50% of technical replicate reactions. Thereby, serial dilutions of the biospecimen are tested and compared to the proportion of positive technical replicate reactions at each dilution^20^. Few studies have performed CSF end-point dilution analysis in a limited number of PD patients^9,21^. So far, results are inconsistent as the authors found a positive association with levels of NFL but not with any clinical measures at baseline and between baseline and Year 3 of the PPMI study. While the PPMI study recruited de-novo patients, the authors also assessed the more advanced BioFIND cohort. While they found an association with age and disease duration, no other clinical markers were associated with end-point dilution^9^. It will be interesting to assess whether higher dilution rates still resulting in a positive seeding response are also associated with the presence/development of cognitive decline. Due to non-linear dynamics in the clinical presentation, continuous clinical measures must be defined as states (e.g., cognitively normal, mild cognitive impairment, dementia).

Additionally, one might argue that differences in seeding activity are linked to conformational seed heterogeneity. Different post-translational modifications of the α-Syn protein potentially caused by differences in cellular pathways engendering different α-Syn strains might modulate their pathogenic effects. A large number of different post-translational modifications, including phosphorylation, nitration, sumoylation, O-GlcNAcylation, and mono- or poly-ubiquitination, have been described for α-Syn^22^. Such post-translational modifications influence the physicochemical properties of the molecule and have been extensively studied as potential biomarkers in PD. Phosphorylation at position 129 (p129- α-Syn), for example, has been shown to be the predominant form of post-translational modifications in Lewy bodies. Different patterns of post-translational modifications could also determine in vitro α-Syn seeding capacity and reflect the biological behavior of the aggregation process, which would reflect the differences in spatiotemporal evolution of the α-Syn pathology in the disease process and, thereby, a different course and spectrum of clinical motor and non-motor manifestations. Indeed, it was already shown that α-Syn strains in people with multisystem atrophy were different and presented with a shorter lag phase and lower Imax in SAA when compared to PD^23^.

Strengths of our study include the large monocentric standardized collection of CSF samples, minimizing variance in sample collection and processing, and the validation of cross-sectional findings in longitudinal cognitive trajectories from the same patients.

We acknowledge the following limitations: (I) We do not present direct evidence that SAA kinetic parameters are caused by different levels of endogenous α-Syn seeds in the reaction or by different α-Syn strains. Thereby, we can not present evidence for the cause underlying our findings of an association between SAA kinetic parameters and the development of cognitive impairment. However, our findings might give ideas for future studies on what to include in analyses beyond a qualitative SAA seeding response. (II) Our definition of cognitive impairment is broad and does not differentiate between normal cognitive status, PD-MCI, and PD dementia based on MDS level II criteria. Moreover, the associations we found between seeding kinetics and cognition are relatively modest and appear to occur relatively late in the disease course. Whether this is due to the global cognitive test used in the study (MoCA instead of comprehensive neuropsychological test battery) or mainly depends on limitations in the precision of current kinetic parameters should be explored in future studies. (III) We did not stratify patients by validated PD subtypes such as the pure motor versus malignant subtype (presence of the combination of cognitive impairment, REM-sleep-behavior disorder, and orthostatic hypotension). (IV) We used different batches of recombinant α-Syn monomer to run the samples, which might have increased the variability between measurements. However, to monitor and limit this potential negative effect, we ran the same negative and positive control in all plates and normalized the relative fluorescent units (RFU) of the tested samples at each time point according to the fluorescence peak reached by the positive control. Notably, the PPMi study used the same batch of recombinant α-Syn monomer for all samples^11^.

We conclude that a prominent CSF α-Syn SAA kinetic profile reflected by a higher number of positive replicates, shorter LAG, higher Imax, and higher AUC is associated with a higher prevalence of cognitive impairment and a shorter duration until the development of cognitive impairment in people with PD.

## Methods

### Participants and clinical investigation

Between 2005 and 2020, 231 PD patients were recruited for lumbar puncture at the University Hospital of Tuebingen. While the cohorts’ mean follow-up was 6 years, the range of follow-up was 1–13 years. All participants were examined by a movement disorders specialist. Diagnosis of PD was defined according to UK Brain Bank Society Criteria^24^. Patients were assessed in dopaminergic ON. We assessed the severity of motor symptoms using part III of the Unified Parkinson’s disease Rating Scale (UPDRS-III), from 2006–2008 the old version, from 2009 on the MDS-UPDRS^25^. The disease stage was categorized by a modified Hoehn and Yahr Scale (H&Y)^26^. Cognitive function was tested with Montreal Cognitive Assessment (MoCA)^27^ and/or Mini Mental Status Examination (MMSE)^28^. Since the MoCA was available only from 2009 on, previously obtained MMSE scores were converted into MoCA equivalents^29^. Cognitive impairment was defined according to criteria reported by Hoops et al. (MoCA ≤25; point of maximum combined sensitivity and specificity)^27^.

The PD patients included in the present study are the same as reported in ref. ^10^. The previous study also included patients with DLB. These have been excluded from the present analysis as they manifest with the key milestone of cognitive impairment already at baseline by definition. While our previous study primarily focussed on the qualitative seeding response across different genetic forms cross-sectionally, here we focused on seeding dynamics represented by the assay’s kinetics in relation to longitudinal clinical slopes. Therefore, only PD patients showing positive seeding activity in the first study were included in the present study. As non-seeders do not show seeding kinetics, these patients have been excluded from the present study.

### Genetic analysis

Genetic screening for mutations in the genes *GBA, LRRK2, parkin,* and *PINK1* was done as previously described^30^. Of the 231 patients, 93 had a *GBA* mutation, 8 had a *LRRK2* mutation, 14 had a *parkin* mutation, 2 had a *PINK1* mutation, and 4 had mutations in two genes. *GBA*-subgroup classification of mutation severity is based on established genotype risks reported for PD (PD_GBA_severe_, PD_GBA_mild_, PD_GBA_risk_)^31^. The supplementary Table 1 lists all different mutations along with the results of CSF RT-QuIC α-Syn seeding per gene and mutation.

### CSF collection

Spinal tap was performed between 9.00 am and 1.00 pm. Samples were centrifuged within 60 min and frozen at −80 °C within 90 min after collection. Samples with abnormal routine CSF diagnostics (erythrocytes >1/μl, white blood cell count >5 cells/μl, immunoglobulin subtype IgG index >0.7) were excluded.

### CSF α-Syn SAA experiments

Purification of recombinant wildtype α-Syn was performed as previously reported^32^, with minor modifications. Transformed *Escherichia coli* BL21 (DE3) bacteria (New England Biolabs) from a glycerol stock were streaked on a selective plate containing kanamycin (Kan+, 50 µg ml^–1^, Sigma) and incubated at 37 °C overnight. A single colony was selected and inoculated into 5 ml of Luria broth (LB, Sigma) with kanamycin and allowed to grow for 4–5 h at 37 °C with continuous agitation at 250 rpm. This starter culture was then added to 1 l of LB containing kanamycin and the overnight express autoinduction system (Merk-Millipore, no. 71300-4) in a fully baffled flask. Cells were grown in a shaking incubator at 37 °C, 200 rpm overnight. The following day the culture was split into four 250 ml flasks and centrifuged at 3200 × *g* for 10 min at 4 °C. The pellet was gently resuspended in 25 ml of osmotic shock buffer containing 40% sucrose (Sigma), 2 mM EDTA (Sigma) and 30 mM Tris (Bio-Rad) at pH 7.2 using a serological pipette, and incubated for 10 min at room temperature under mild agitation on a rotator mixer. The solution was then centrifuged at 9000 × *g*, 20 min at 20 s and 20 µl of saturated MgCl_2_ (Sigma) added. After 3 min incubation under mild rocking on ice the suspension was centrifuged at 9000 × *g* for 30 min at 4 °C, and the supernatant collected into a 100 ml glass beaker. pH was reduced to 3.5 by the addition of 400–600 µl HCl 1 M (PanReac AppliChem) and incubated under stirring for 10 min at room temperature. After a second centrifugation at 9000 × *g* for 30 min at 4 °C, the supernatant was collected into a clean 100 ml glass beaker. pH was adjusted to 7.5 by the addition of 400–600 µl of NaOH 1 M (Sigma). The protein extract was filtered through a 0.22 µm filter (Merk-Millipore), loaded into a Ni–NTA column (Cytiva, no. 17525501) on an NGC chromatography system (Bio-Rad) and washed with 20 mM Tris pH 7.5 at room temperature. The column was further washed with 50 mM imidazole (Sigma) in Tris 20 mM pH 7.5, generating a peak that was not collected. A linear gradient up to 500 mM imidazole in 20 mM Tris pH 7.5 was performed, and the peak was collected between 30 and 75% of imidazole buffer (150 and 375 mM, respectively). This peak was loaded onto a Q-HP anion exchange column (Cytiva, no. 17115401) and washed in Tris 20 mM pH 7.5, followed by another washing in 100 mM NaCl in Tris 20 mM pH 7.5. Again, a linear gradient up to 500 mM of NaCl in Tris 20 mM pH 7.5 was carried out to collect the peak between 300 and 350 mM NaCl. The fractions were pooled, filtered through a 0.22 µm filter, and dialyzed against Milli-Q water overnight at 4 °C using a 3.5 kDa MWCO dialysis membrane (Thermo-Scientific). The following day, the protein was moved into fresh Milli-Q water and dialyzed for a further 4 h. Protein concentration was measured by spectrophotometry using a theoretical extinction coefficient at 280 nm of 0.36 (mg/ml)^−1^ cm^−1^, and the final solution containing the purified α-Syn was aliquoted in order to have 0.5 mg of α-Syn per tube. Finally, the protein was lyophilized for 6 h and stored at −80 °C until use. The recombinant α-Syn was eventually resuspended into 0.5 ml of phosphate buffer (PB, 40 mM, pH 8.0, Sigma) immediately before the execution of the α-Syn SAA.

CSF α-Syn seed amplification experiments were performed at the Laboratory of Neuropathology of the Institute of Neurological Science of Bologna (LabNP at ISNB). The assay showed a high specificity (98.7%) for Lewy body pathology in a series of 121 CSF samples from individuals referred to the LabNP at ISNB for dementia of various aetiologies in which the presence of Lewy-related abnormal α-Syn deposits was excluded by neuropathological examination^31^.

Briefly, six 0.8 mm silica beads (OPS Diagnostics) per well were preloaded into black, clear-bottom, 96-well plates (Nalgene Nunc International). CSF samples were thawed and vortexed 10 s before use. Fifteen microliters of CSF were added to 85 μl of a reaction mix composed of 40 mM PB pH 8.0, 170 mM NaCl, 10 mM thioflavin-T (Sigma), 0.0015% SDS (Bio-Rad), and 0.1 g l^–1^ filtered recombinant α-Syn (100 kDa Amicon centrifugal filters, Merck Millipore). Plates were closed with a plate sealer film (Nalgene Nunc International) and incubated into a Fluostar Omega plate reader (BMG Labtech) at 42 °C with intermittent double-orbital shaking at 400 rpm for 1 min, followed by 1 min rest. Fluorescence was measured every 45 min with 450 nm excitation and 480 nm emission filter. In addition to a negative control, we ran the same positive sample throughout all experiments to optimize the comparison between fluorescent responses in different plates. As negative controls, we used CSF samples from patients with normal pressure hydrocephalus, which gave a negative result in two RT-QuIC runs. The positive control was a CSF sample from a patient with normal pressure hydrocephalus, which gave at least 3 of 4 positive replicates in two RT-QuIC runs. To overcome batch-to-batch variations and intrinsic plate-to-plate variability, we normalized within each plate the relative fluorescent units (RFU) of the tested samples at each time point according to the fluorescence peak reached by the positive control by expressing the RFU values as percentages of the maximum fluorescence intensity (i.e., highest RFU value) of the positive control, which we considered 100% (Supplementary Table 6, Supplementary Figure 1).

Each CSF sample was run in quadruplicate and deemed positive when at least 2 out 4 replicates crossed the threshold. We calculated the threshold as the average normalized fluorescence value of negative control repeats during the first 10 h of recording plus 30 standard deviations. The cut-off was set at 30 h. When only one of the four replicates crossed the threshold, the analysis was considered “unclear” and repeated up to three times. In those participants who showed a positive CSF α-Syn seeding profile, we measured the individual mean LAG (time required to reach the threshold; mean out of all positive replicates), the individual mean peak of the fluorescence response (Imax, mean out of all positive replicates), and the individual mean area under the curve (AUC; mean out of all positive replicates). Inter-assay coefficients of variability were <12% for all three kinetic parameters (LAG 7.7%, Imax 6.8%, AUC 11.8%).

All experiments were done, and results were reported blinded to the clinical diagnosis and genetic status.

### CSF measurement of Amyloid-β_1‑42_, total‑Tau, phospho181‑Tau, neurofilament light chain protein (NFL) and total α-Syn

CSF levels of Amyloid-β_1–42_, total-Tau, and phospho181-Tau were measured using ELISA kits from INNOTEST, Fujirebio GmbH, Germany. Our validated in-house cut-offs for clinical routine indicating pathological levels are as follows: Amyloid-β_1–42_ ≤ 600 pg/ml, total-Tau >450 pg/ml, phospho181-Tau >60 pg/ml. We used these cut-offs to define an Alzheimer’s Disease CSF profile according to the National Institute on Aging (NIA)-Alzheimer’s Association (AA) Research Framework. CSF levels of NFL were measured using the Uman Diagnostics NF-Light assay. CSF levels of total α-Syn were assessed using an ELISA kit for human α-synuclein (Roboscreen GmbH, Germany).

Intra-assay coefficients of variation for Amyloidβ_1–42_, total-Tau, phospho181-Tau, and NFL were below 15% and for total α-synuclein below 5%.

### Statistical analysis

Statistical analysis was performed using IBM SPSS 26.0 software. All patients included in the present analyses were clearly CSF α-Syn seeding positive. They were stratified into subgroups based on their number of positive CSF α-Syn seeding replicates and into tertiles based on their individual CSF α-Syn seeding kinetic parameters (LAG, Imax, AUC, respectively). We opted for tertiles to maintain enough power per subgroup. For LAG, the highest tertile means a long LAG phase, whereas the lowest tertile means a short LAG phase. For AUC and Imax, the highest tertile means high AUC and high Imax, respectively.

Group comparisons of dichotomous data were analyzed using the likelihood-ratio chi-square test. Correlation analyses between the number of positive CSF α-Syn seeding replicates and CSF α-Syn seeding kinetic parameters with demographic/clinical measures were performed by using Spearman-Rho. Intergroup comparisons stratified by the number of positive α-Syn seeding replicates and by kinetic tertiles (AUC, Imax, LAG, respectively) were calculated using ANOVA/ANCOVA with age and disease duration as co-variates where appropriate. In case of significant differences in comparisons of more than two groups, post-hoc Bonferroni subgroup comparison was performed for continuous data.

The effect of CSF α-Syn kinetic parameters on the longitudinal development of cognitive impairment was analyzed by Cox-Regression with baseline MoCA and AUC, Imax, LAG tertiles as interacting co-variates.

Hypothesis testing was 2-sided and p values ≤ 0.05 were considered statistically significant.

### Supplementary information

Supplementary Material

nr-reporting-summary

## Data Availability

Anonymized data are available upon request to: kathrin.brockmann@uni-tuebingen.de.
