# Supplementary material for: CSF α-synuclein seed amplification kinetic profiles are associated with cognitive decline in Parkinson’s disease
Source: NPJ Parkinsons Dis. 2024 Jan 20;10:24. doi: 10.1038/s41531-023-00627-5 (PMC10799016; doi:10.1038/s41531-023-00627-5)
Supplement: Supplementary file 1 — Supplementary Material [file 41531_2023_627_MOESM1_ESM.pdf]

Supplementary Tables 1: Demographic, clinical and CSF characteristics stratified by number of positive seeding replicates for PD-wildtype and PD-GBA separately

**Number of positive seeding replicates out of 4 (PD-Wildtype)**	**2**	**3**	**4**	**p-value**
Male sex %	100	52	77	0.002
Age, years	59 ± 8	62 ± 8	68 ± 8 ***	≤0.001
Age at onset, years	55 ± 7	58 ± 8	62 ± 8 *	0.004
Disease duration, years	4 ± 3	4 ± 3	5 ± 3	0.145
H&Y	2.0 ± 0.3	2.0 ± 0.5	2.0 ± 0.6	0.407
H&Y ≥2.5 %	14	3	9	0.409
UPDRS-III	22 ± 6	21 ± 10	26 ± 10	0.135
Montreal Cognitive Assessment	27 ± 2	27 ± 4	25 ± 4 *	0.031
Cognitive impairment<br>- At baseline %<br>- New during study %<br>- Cognitive impairment anytime %	 23<br>20<br>39	 17<br>28<br>40	 36<br>19<br>48	 0.158<br>0.718<br>0.687
BDI-II	9 ± 10	7 ± 6	8 ± 7	0.542
LEDD	370 ± 246	355 ± 311	416 ± 265	0.589
Amyloidβ$_{1-42}$ [pg/ml]	748 ± 213	686 ± 175	659 ± 277	0.463
total-Tau [pg/ml]	176 ± 68	206 ± 105	245 ± 132	0.089
phospho181-Tau [pg/ml]	32 ± 9	39 ± 15	42 ± 15	0.068
AD Profile %	0	0	1.8	0.671
Neurofilament light chain [pg/ml]	854 ± 1132	805 ± 483	899 ± 421	0.775
total α-synuclein [pg/ml]	527 ± 253	527 ± 214	598 ± 290	0.409
LAG, h	22 ± 3	22 ± 3	21 ± 3 * §	0.034
Imax	61 ± 14	65 ± 14	71 ± 13 ** §	0.019
AUC	605 ± 207	658 ± 217	770 ± 231 ** §	0.014

**Number of positive seeding replicates out of 4 (PD-GBA)**	**2**	**3**	**4**	**p-value**
Male sex %	0	68	66	0.059
Age, years	57 ± 14	61 ± 10	64 ± 9	0.264
Age at onset, years	53 ± 16	54 ± 11	55 ± 9	0.846
Disease duration, years	3 ± 3	7 ± 4	9 ± 6	0.185
H&Y	2.0 ± 0.0	2.1 ± 0.4	2.3 ± 0.8	0.566
H&Y ≥2.5 %	0	16	29	0.293
UPDRS-III	20 ± 6	29 ± 9	28 ± 12	0.427
Montreal Cognitive Assessment	23 ± 10	27 ± 3	23 ± 5 §§	0.016
Cognitive impairment<br>- At baseline %<br>- New during study %<br>- Cognitive impairment anytime %	 33<br>0<br>33	 16<br>31<br>42	 48<br>30<br>64	 0.045<br>0.647<br>0.175
BDI-II	7 ± 8	9 ± 5	10 ± 7	0.511
LEDD	437 ± 28	555 ± 330	662 ± 442	0.440
Amyloidβ_{1-42} [pg/ml]	783 ± 95	727 ± 232	729 ± 283	0.943
total-Tau [pg/ml]	216 ± 91	225 ± 113	251 ± 140	0.717
phospho181-Tau [pg/ml]	39 ± 15	35 ± 10	40 ± 14	0.436
AD Profile %	0	0	0	n.a.
Neurofilament light chain [pg/ml]	838 ± 576	699 ± 262	1078 ± 1261	0.417
total α-synuclein [pg/ml]	498 ± 162	529 ± 296	545 ± 222	0.923
LAG, h	23 ± 1	21 ± 3	19 ± 3 * §	0.007
Imax	61 ± 8	70 ± 13	72 ± 13	0.245
AUC	569 ± 60	733 ± 262	837 ± 214 *	0.040

BDI-II = Becks depression Inventory version II; H&Y = Hoehn and Yahr; LEDD = Levodopa Equivalent Daily Dose; UPDRS-III = Unified Parkinson Disease rating Scale part III.

Data are presented as mean and standard deviation.

Post-hoc Bonferroni subgroup comparison for continuous data 2 vs. 4 levels of significance: * p<0.05; **p<0.01; ***p≤0.001.

Post-hoc Bonferroni subgroup comparison for continuous data 3 vs. 4 levels of significance: § p<0.05; §§ p<0.01; §§§ p≤0.001.

Supplementary Tables 2: Demographic, clinical and CSF characteristics stratified by LAG Tertile groups for PD-wildtype and PD-GBA separately

LAG<br><br>**PD-Wildtype**	**Highest Tertile (longest LAG)**	**Mid Tertile**	**Lowest tertile (shortest LAG)**	**p-value**
Male sex %	73	68	79	0.608
Age, years	64 ± 8	65 ± 10	66 ± 7	0.687
Age at onset, years	60 ± 8	60 ± 9	61 ± 7	0.883
Disease duration, years	5 ± 3	5 ± 3	5 ± 3	0.457
H&Y	1.9 ± 0.5	1.9 ± 0.4	2.0 ± 0.7	0.460
H&Y ≥2.5 %	7	8	8	0.986
UPDRS-III	24 ± 9	22 ± 10	25 ± 11	0.602
Montreal Cognitive Assessment	26 ± 3	26 ± 4	25 ± 4	0.641
Cognitive impairment<br>- At baseline %<br>- New during study %<br>- Cognitive impairment anytime %	 28<br>17<br>40	 28<br>19<br>42	 30<br>38<br>57	 0.966<br>0.262<br>0.408
BDI-II	8 ± 7	8 ± 9	7 ± 5	0.813
LEDD	456 ± 303	347 ± 261	351 ± 242	0.164
Amyloidβ_{1-42} [pg/ml]	680 ± 205	687 ± 254	668 ± 287	0.956
total-Tau [pg/ml]	232 ± 158	220 ± 91	215 ± 74	0.828
phospho181-Tau [pg/ml]	40 ± 18	38 ± 13	39 ± 10	0.807
AD Profile %	2.4	0	0	0.472
Neurofilament light chain [pg/ml]	813 ± 705	949 ± 571	810 ± 283	0.531
total α-synuclein [pg/ml]	581 ± 264	598 ± 309	491 ± 158	0.280

LAG<br><br>**PD-GBA**	**Highest Tertile (longest LAG)**	**Mid Tertile**	**Lowest tertile (shortest LAG)**	**p-value**
Male sex %	74	48	70	0.102
Age, years	65 ± 11	64 ± 10	61 ± 8	0.227
Age at onset, years	58 ± 12	55 ± 9	53 ± 8	0.257
Disease duration, years	8 ± 5	9 ± 7	8 ± 5	0.400
H&Y	2.2 ± 0.8	2.4 ± 0.8	2.1 ± 0.6	0.201
H&Y ≥2.5 %	22	33	22	0.511
UPDRS-III	25 ± 11	28 ± 11	29 ± 12	0.472
Montreal Cognitive Assessment	25 ± 5	23 ± 6	25 ± 4	0.502
Cognitive impairment<br>- At baseline %<br>- New during study %<br>- Cognitive impairment anytime %	 36<br>14<br>46	 44<br>40<br>67	 39<br>32<br>58	 0.834<br>0.299<br>0.325
BDI-II	11 ± 6	10 ± 6	10 ± 7	0.758
LEDD	593 ± 340	726 ± 497	589 ± 392	0.393
Amyloidβ_{1-42} [pg/ml]	714 ± 231	779 ± 266	708 ± 291	0.566
total-Tau [pg/ml]	294 ± 214	258 ± 79	204 ± 74 *	0.030
phospho181-Tau [pg/ml]	39 ± 11	47 ± 15	33 ± 11°°°	≤0.001
AD Profile %	0	0	0	n.a.
Neurofilament light chain [pg/ml]	1387 ± 1937	869 ± 404	813 ± 556	0.122
total α-synuclein [pg/ml]	543 ± 191	585 ± 286	503 ± 221	0.411

BDI-II = Becks depression Inventory version II; H&Y = Hoehn and Yahr; LEDD = Levodopa Equivalent Daily Dose; UPDRS-III = Unified Parkinson Disease rating Scale part III.

Data are presented as mean and standard deviation.

Post-hoc Bonferroni subgroup comparison for continuous data shortest vs. mid Tertile with levels of significance: ° $p<0.05$; °° $p<0.01$; °°° $p\leq0.001$.

Post-hoc Bonferroni subgroup comparison for continuous data shortest vs. longest Tertile levels of significance: * $p<0.05$; ** $p<0.01$; *** $p\leq0.001$.

Supplementary Tables 3: Demographic, clinical and CSF characteristics stratified by Imax Tertile groups for PD-wildtype and PD-GBA separately

Imax<br><br>PD-Wildtype	Lowest Tertile (lowest Imax)	Mid Tertile	Highest Tertile (highest Imax)	p-value
Male sex %	69	82	69	0.410
Age, years	64 ± 7	66 ± 10	65 ± 9	0.495
Age at onset, years	59 ± 7	61 ± 9	60 ± 9	0.645
Disease duration, years	5 ± 3	5 ± 3	5 ± 3	0.707
H&Y	1.9 ± 0.4	1.9 ± 0.4	2.0 ± 0.7	0.533
H&Y ≥2.5 %	7	7	9	0.927
UPDRS-III	24 ± 9	24 ± 10	24 ± 11	0.981
Montreal Cognitive Assessment	27 ± 3	25 ± 5	26 ± 3	0.124
Cognitive impairment<br>- At baseline %<br>- New during study %<br>- Cognitive impairment anytime %	 20<br>15<br>32	 39<br>24<br>54	 30<br>33<br>53	 0.195<br>0.295<br>0.100
BDI-II	8 ± 7	8 ± 7	8 ± 7	0.970
LEDD	399 ± 309	471 ± 287	311 ± 198	0.078
Amyloidβ_{1-42} [pg/ml]	700 ± 191	682 ± 266	651 ± 280	0.690
total-Tau [pg/ml]	229 ± 139	231 ± 126	210 ± 81	0.732
phospho181-Tau [pg/ml]	40 ± 15	40 ± 17	38 ± 12	0.829
AD Profile %	2.4	0	0	0.486
Neurofilament light chain [pg/ml]	843 ± 738	940 ± 536	823 ± 332	0.724
total α-synuclein [pg/ml]	601 ± 295	573 ± 225	515 ± 252	0.394

Imax<br><br>PD-GBA	Lowest Tertile (lowest Imax)	Mid Tertile	Highest Tertile (highest Imax)	p-value
Male sex %	61	72	59	0.533
Age, years	61 ± 10	63 ± 11	65 ± 6	0.193
Age at onset, years	53 ± 10	55 ± 11	57 ± 8	0.218
Disease duration, years	8 ± 6	8 ± 6	8 ± 5	0.996
H&Y	2.0 ± 0.8	2.3 ± 0.6	2.3 ± 0.8	0.251
H&Y ≥2.5 %	13	25	34	0.199
UPDRS-III	25 ± 10	28 ± 12	30 ± 12	0.248
Montreal Cognitive Assessment	26 ± 3	23 ± 6	24 ± 5	0.086
Cognitive impairment<br>- At baseline %<br>- New during study %<br>- Cognitive impairment anytime %	 19<br>24<br>38	 56<br>14<br>63	 38<br>45<br>66	 0.024<br>0.124<br>0.109
BDI-II	10 ± 6	11 ± 7	9 ± 7	0.357
LEDD	636 ± 525	664 ± 374	591 ± 364	0.785
Amyloidβ_{1-42} [pg/ml]	814 ± 275	702 ± 267	697 ± 256	0.213
total-Tau [pg/ml]	274 ± 188	248 ± 129	218 ± 73	0.301
phospho181-Tau [pg/ml]	40 ± 11	41 ± 17	36 ± 12	0.402
AD Profile %	0	0	0	n.a.
Neurofilament light chain [pg/ml]	1164 ± 1957	936 ± 503	900 ± 571	0.662
total α-synuclein [pg/ml]	575 ± 203	560 ± 261	497 ± 235	0.423

BDI-II = Becks depression Inventory version II; H&Y = Hoehn and Yahr; LEDD = Levodopa Equivalent Daily Dose; UPDRS-III = Unified Parkinson Disease rating Scale part III.

Data are presented as mean and standard deviation.

Supplementary Tables 4: Demographic, clinical and CSF characteristics stratified by AUC Tertile groups for PD-wildtype and PD-GBA separately

Area under the curve (AUC)<br><br>**PD-Wildtype**	Lowest Tertile (lowest AUC)	Mid Tertile	Highest Tertile (highest AUC)	p-value
Male sex %	71	72	76	0.889
Age, years	64 ± 8	65 ± 10	66 ± 8	0.835
Age at onset, years	60 ± 8	60 ± 9	61 ± 8	0.842
Disease duration, years	5 ± 3	5 ± 3	5 ± 3	0.946
H&Y	1.9 ± 0.4	2.0 ± 0.4	2.0 ± 0.7	0.473
H&Y ≥2.5 %	5	13	7	0.474
UPDRS-III	24 ± 9	23 ± 10	25 ± 11	0.762
Montreal Cognitive Assessment	27 ± 3	25 ± 4	26 ± 3	0.214
Cognitive impairment<br>- At baseline %<br>- New during study %<br>- Cognitive impairment anytime %	 20<br>19<br>35	 47<br>18<br>56	 19<br>32<br>44	 0.018<br>0.454<br>0.197
BDI-II	9 ± 7	7 ± 8	7 ± 6	0.385
LEDD	432 ± 317	390 ± 250	335 ± 242	0.355
Amyloid$\beta_{1\text{-}42}$ [pg/ml]	696 ± 192	652 ± 255	686 ± 291	0.733
total-Tau [pg/ml]	237 ± 141	214 ± 122	216 ± 75	0.665
phospho181-Tau [pg/ml]	42 ± 16	37 ± 16	39 ± 10	0.368
AD Profile %	2.4	0	0	0.472
Neurofilament light chain [pg/ml]	803 ± 690	995 ± 612	796 ± 292	0.302
total α-synuclein [pg/ml]	618 ± 286	559 ± 297	502 ± 166	0.203

Area under the curve (AUC)<br><br>**PD-GBA**	Lowest Tertile (lowest AUC)	Mid Tertile	Highest Tertile (highest AUC)	p-value
Male sex %	71	58	66	0.600
Age, years	63 ± 10	64 ± 10	63 ± 9	0.958
Age at onset, years	56 ± 11	54 ± 10	55 ± 9	0.853
Disease duration, years	7 ± 4	9 ± 7	8 ± 5	0.349
H&Y	2.1 ± 0.8	2.3 ± 0.6	2.2 ± 0.7	0.626
H&Y ≥2.5 %	19	26	29	0.727
UPDRS-III	25 ± 11	28 ± 11	29 ± 12	0.409
Montreal Cognitive Assessment	25 ± 5	23 ± 5	25 ± 5	0.287
Cognitive impairment<br>- At baseline %<br>- New during study %<br>- Cognitive impairment anytime %	 30<br>14<br>40	 50<br>40<br>70	 37<br>32<br>57	 0.332<br>0.299<br>0.109
BDI-II	11 ± 7	10 ± 5	10 ± 7	0.753
LEDD	615 ± 346	640 ± 468	632 ± 413	0.977
Amyloid$\beta_{1\text{-}42}$ [pg/ml]	739 ± 226	773 ± 279	690 ± 281	0.461
total-Tau [pg/ml]	267 ± 199	269 ± 121	209 ± 77	0.130
phospho181-Tau [pg/ml]	40 ± 10	44 ± 16	34 ± 12 §	0.015
AD Profile %	0	0	0	n.a.
Neurofilament light chain [pg/ml]	1403 ± 2032	848 ± 409	847 ± 557	0.135
total α-synuclein [pg/ml]	550 ± 176	571 ± 273	507 ± 181	0.557

BDI-II = Becks depression Inventory version II; H&Y = Hoehn and Yahr; LEDD = Levodopa Equivalent Daily Dose; UPDRS-III = Unified Parkinson Disease rating Scale part III.

Data are presented as mean and standard deviation.

Post-hoc Bonferroni subgroup comparison for continuous data highest vs. mid Tertile level of significance: § $p<0.05$; §§ $p<0.01$; §§§ $p\leq0.001$.

Supplementary Table 5: Overview of Genetic Variants and CSF α-Syn seeding results of the genetic cases

**GBA**<br>**n=93**	**LRKK2**<br>**n=8**	**PRKN**<br>**n=14**	**PINK1**<br>**n=2**	**Mutations in > 1 genes**<br>**n=4**
L444P (GBA$_{severe}$)<br>(n=14, seeding pos n=14)	G2019S<br>(n=4, seeding pos n=4)	P437L<br>heterozygous<br>(n=3, seeding pos n=0)	Q126P<br>homozygous<br>(n=2, seeding pos n=0)	I2020T (LRRK2) + Y304C (GBA$_{severe}$)<br>(n=1, seeding pos n=0)
c.115+1G>A (GBA$_{severe}$)<br>(n=3, seeding pos n=3)	R1441C<br>(n=2, seeding pos n=2)	R275W<br>heterozygous<br>(n=3, seeding pos n=3)		ex3+4del (PRKN) + T369M (GBA$_{risk}$)<br>(n=1, seeding pos n=1)
W184R (GBA$_{severe}$)<br>(n=2, seeding pos n=2)	N1437S<br>(n=1, seeding pos n=1)	R234Q<br>heterozygous<br>(n=1, seeding pos n=1)		ex4del; ex8+9del (PRKN) + H255Q (GBA$_{severe}$)<br>(n=1, seeding pos n=0)
L444P, T369M (GBA$_{severe}$)<br>(n=1, seeding pos n=1)	I2020T<br>(n=1, seeding pos n=0)	ex2del<br>heterozygous<br>(n=1, seeding pos n=1)		R275W (PRKN) + S271G (GBA$_{mild}$)<br>(n=1, seeding pos n=1)
L444P, E388K (GBA$_{severe}$)<br>(n=1, seeding pos n=1)		ex2dup<br>heterozygous<br>(n=1, seeding pos n=1)		
L444P, E326K, N392S (GBA$_{severe}$)<br>(n=1, seeding pos n=1)		ex3+4del<br>heterozygous<br>(n=1, seeding pos n=1)		
L444P, E326K (GBA$_{severe}$)<br>(n=1, seeding pos n=1)		ex8+9del<br>heterozygous<br>(n=1, seeding pos n=1)		
c.1265_1319del, D409H, L444P (GBA$_{severe}$)<br>(n=1, seeding pos n=1)		c.101_102delAG;<br>ex3+4del<br>bi-allelic<br>(n=1, seeding pos n=0)		
D409H (GBA$_{severe}$)<br>(n=1, seeding pos n=1)		ex2del; ex7dup<br>bi-allelic<br>(n=1, seeding pos n=0)		
G202R (GBA$_{severe}$)<br>(n=1, seeding pos n=1)		ex3+4+5+6del<br>homozygous<br>(n=1, seeding pos n=0)		
R359X (GBA$_{severe}$)<br>(n=1, seeding pos n=1)				
N370S (GBA$_{mild}$)<br>(n=14, seeding pos n=9)				
D140H, E326K (GBA$_{mild}$)<br>(n=2, seeding pos n=2)				
S271G (GBA$_{mild}$)<br>(n=1, seeding pos n=1)				
S271G, N370S (GBA$_{mild}$)<br>(n=1, seeding pos n=1)				
E326K (GBA$_{risk}$)<br>(n=33, seeding pos n=30)				
T369M (GBA$_{risk}$)<br>(n=13, seeding pos n=13)				
R39C (GBA$_{risk}$)<br>(n=1, seeding pos n=1)				
T297S (GBA$_{risk}$)<br>(n=1, seeding pos n=0)				

n=: total sample size per mutation
seeding pos n=: number of patients with positive CSF α-Syn seeding out of the total sample size per mutation

Supplementary Table 6: CSF α-Syn seeding kinetic raw data for the total cohort and stratified for PD-wildtype and PD-GBA separately

	Total cohort	PD wildtype	PD GBA
**LAG raw**	19.9	20.5	19.2
**Imax raw**	121303	121513	121162
**AUC raw**	1301025	1265601	1342378

Supplementary Figure 1: Representation of raw fluorescence kinetic signals of α-Syn SAA.

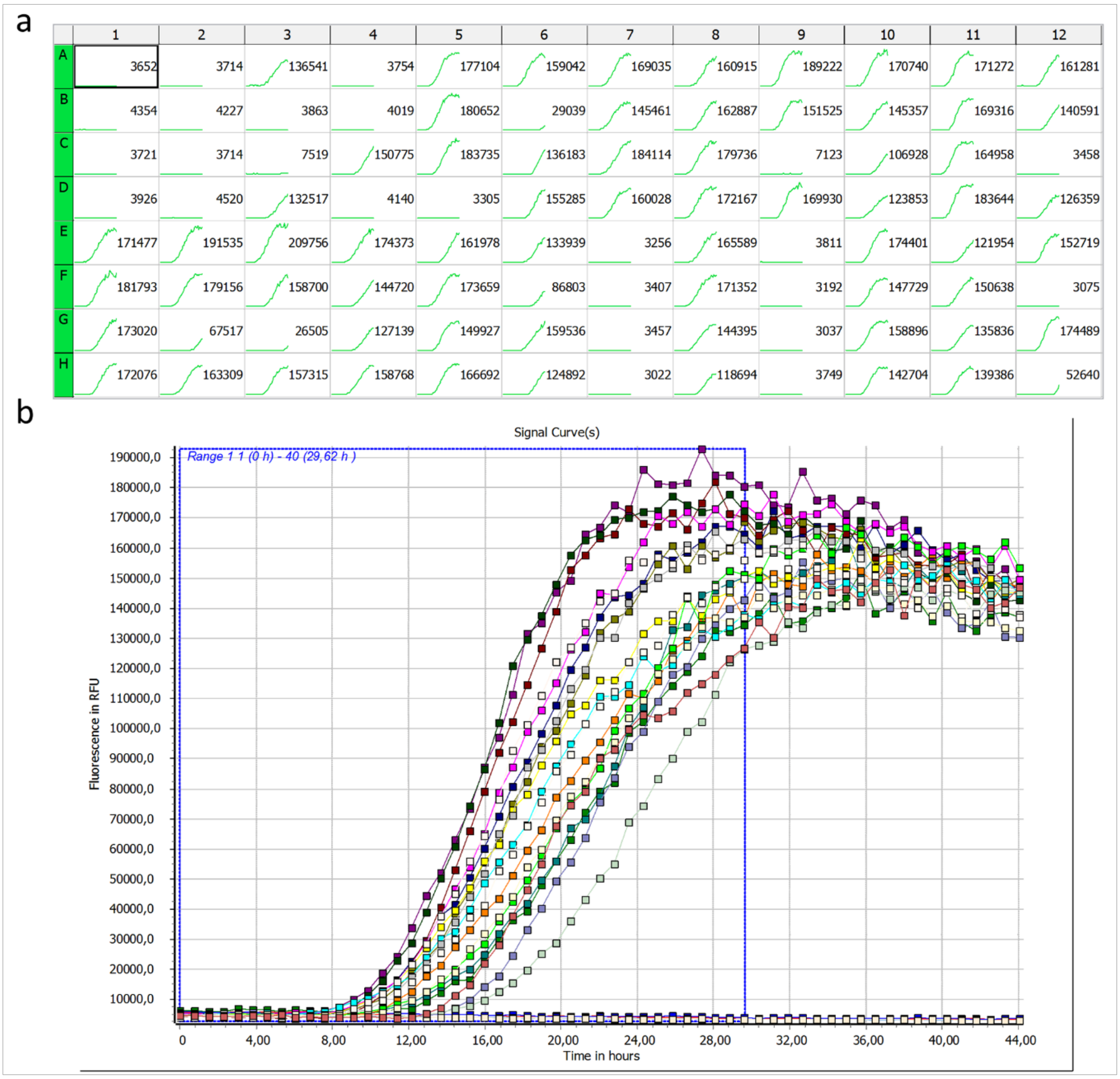

(a) Representative results of a 96-well plate run including one negative (lane 1, top) and positive (lane 1, bottom) control samples and 20 patients (lanes 2-11). (b) Kinetic curves are shown as the average of the positive replicates of each sample. The “unclear” sample (1 out of 4 positive replicates) in lane 4 was excluded.
